# Supplementary figures and images for: Intranasal exposure of African green monkeys to SARS-CoV-2 results in acute phase pneumonia with shedding and lung injury still present in the early convalescence phase
Source: Res Sq. 2020 Aug 13:rs.3.rs-50023. Preprint. [Version 2] doi: 10.21203/rs.3.rs-50023/v2 (PMC7430587; doi:10.21203/rs.3.rs-50023/v2)

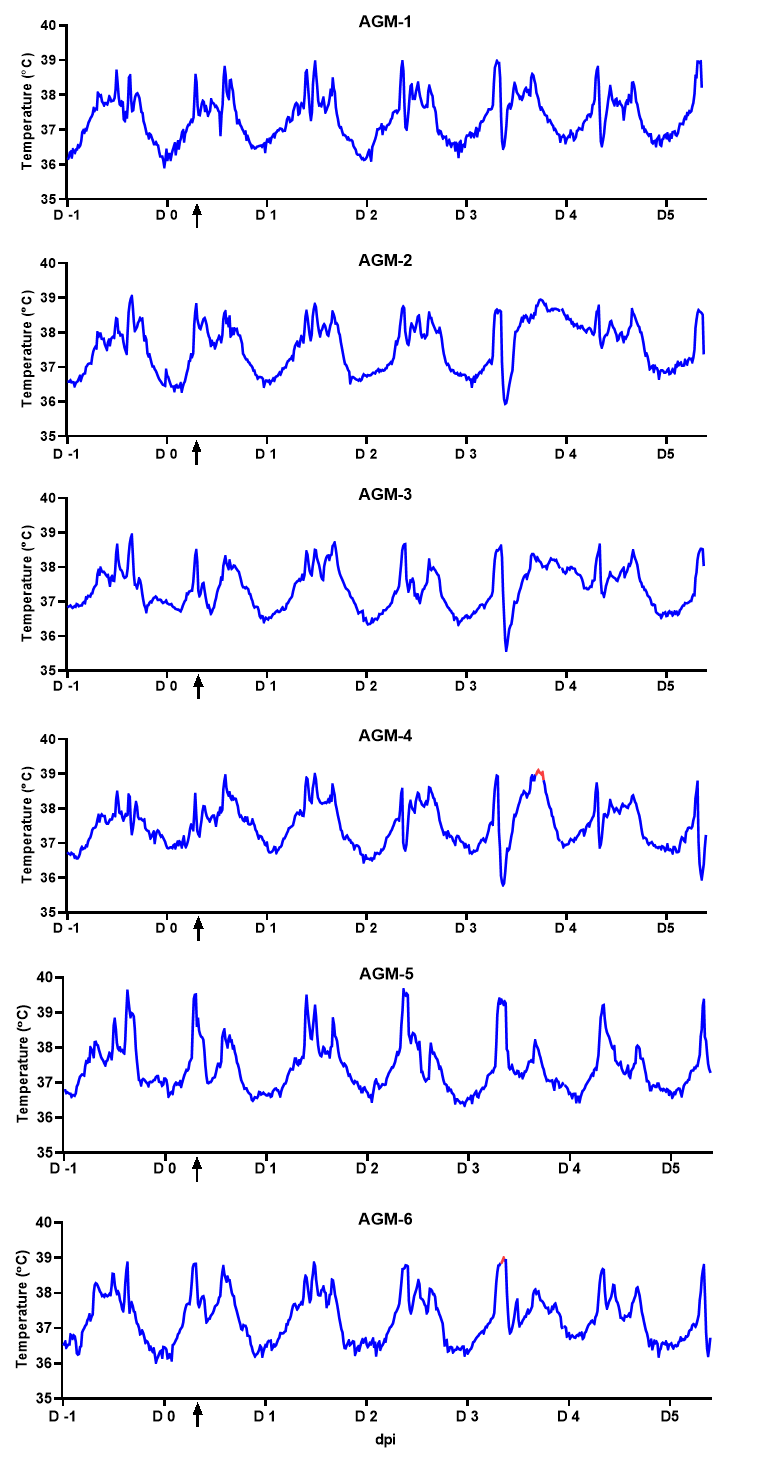

Supplement: Supplement [file SUPPLEMENTARYFIGURE1TEMPERATURE.tif]
