# Supplementary material for: Intranasal exposure of African green monkeys to SARS-CoV-2 results in acute phase pneumonia with shedding and lung injury still present in the early convalescence phase
Source: Res Sq. 2020 Aug 13:rs.3.rs-50023. Preprint. [Version 2] doi: 10.21203/rs.3.rs-50023/v2 (PMC7430587; doi:10.21203/rs.3.rs-50023/v2)
Supplement: Supplement [file SupplemenaryTable1.docx]

**Supplementary Table 1. Clinical description and outcome of African green monkeys following SARS-CoV-2 challenge**

| **Subject No.** | **Sex** | **Clinical illness** | **Clinical pathology** |
| --- | --- | --- | --- |
| AGM-1 | F | Decreased appetite (d0-5). Subject survived to study endpoint (d5). | Lymphocytopenia (d2, 3, 5); monocytopenia (d2, 3, 5); neutropenia (d2); eosinopenia (d2, 3); neutrophilia (d4); eosinophilia (d4); basophilia (d4, 5); hypoglycemia (d2, 4); > 4-fold ↑ CRP (d4); hypercapnia (d3-5) |
| AGM-2 | F | Decreased appetite (d0-3, 5); anorexia (d4). Subject survived to study endpoint (d5). | Lymphocytopenia (d2-5); eosinopenia (d2, 3, 5); basopenia (d2, 3, 5); thrombocytopenia (d4); neutrophilia (d4); eosinophila (d4); basophilia (d4); hypoglycemia (d2-5); > 1-fold ↑ CRE (d5); hypoamylasemia (d4); > 16-fold ↑ CRP (d4) |
| AGM-3 | F | Decreased appetite (d0-3, 5); anorexia (d4). Subject survived to study endpoint (d5). | Lymphocytopenia (d2, 3, 5); neutropenia (d2); eosinopenia (d3); basopenia (d3); monocytosis (d4); neutrophilia (d4); eosinophilia (d4, 5); basophilia (d4, 5); > 4-fold ↑ CRP (d4) |
| AGM-4 | F | Decreased appetite (d0-1, 3-6, 8, 14-16, 19, 22, 29). Subject survived to study endpoint (d34). | Lymphocytopenia (d2-5, 7, 12); monocytopenia (d2-5, 12, 15, 34); neutropenia (d2); eosinopenia (d2); basopenia (d2); neutrophilia (d4, 5, 7, 9, 12, 15); eosinophilia (d4, 9, 12, 15, 21, 34); basophilia (d4, 9, 12, 15, 21, 34); hypoglycemia (d2-5, 7, 9, 12, 15, 21, 28, 34); > 4-fold ↑ CRP (d4); hypercapnia (d4, 5, 7, 15, 21, 28) |
| AGM-5 | F | Decreased appetite (d0-1, 3-8, 12-14, 17, 22, 23, 26-29, 31, 33). Subject survived to study endpoint (d34). | Basopenia (d2,3); monocytosis (d12, 34); neutrophilia (d34); eosinophilia (d34); basophilia (9, 12, 15, 21, 34); hypoglycemia (d2-5, 12, 28, 34); > 1-fold ↑ ALT (d34); hypercapnia (d5, 7, 21, 28, 34) |
| AGM-6 | F | Decreased appetite (d0-8, 10, 12, 13, 15, 16, 18, 22, 23, 28, 29, 34). Subject survived to study endpoint (d34). | Lymphocytopenia (d2,3); eosinopenia (d3, 28); basopenia (d28); monocytosis (d2-4, 9, 12); neutrophilia (d5, 7, 9, 12, 21, 34); eosinophilia (d9, 12, 21, 34); basophilia (d9, 12, 15, 21, 34); hypoglycemia (d2); ≥ 2-fold ↑ ALT (d21, 28, 34); > 1-fold ↑ CRP (d4) |
|  |  |  |  |

Days after SARS-CoV-2 challenge are in parentheses. All reported findings are in comparison to baseline (d0) values. Decreased appetite is defined as some food but not all food consumed from the previous day. Anorexia is defined as no food consumed from the previous day. Lymphocytopenia, monocytopenia, erythrocytopenia, thrombocytopenia, neutropenia, eosinopenia, and basopenia are defined by a ≥35% drop in numbers of lymphocytes, monocytes, erythrocytes, platelets, neutrophils, eosinophils, and basophils, respectively. Lymphocytosis, monocytosis, neutrophilia, eosinophilia, and basophilia are defined by a 100% or greater increase in numbers of lymphocytes, monocytes, neutrophils, eosinophils, or basophils, respectively. Hyperglycemia is defined as a 100% or greater increase in levels of glucose. Hypoglycemia is defined by a ≥25% decrease in levels of glucose. Hypoalbuminemia is defined by a ≥25% decrease in levels of albumin. Hypoproteinemia is defined by a ≥25% decrease in levels of total protein. Hypoamylasemia is defined by a ≥25% decrease in levels of serum amylase. Hypocalcemia is defined by a ≥25% decrease in levels of serum calcium. Hypercapnia was defined as having a partial CO2 >4 mmHg over d0 baseline values. (ALT) alanine aminotransferase, (AST) aspartate aminotransferase, (ALP) alkaline phosphatase, (CRE) Creatinine, (CRP) C-reactive protein, (Hct) hematocrit, (Hgb) hemoglobin.
