# Supplementary material for: Intranasal exposure of African green monkeys to SARS-CoV-2 results in acute phase pneumonia with shedding and lung injury still present in the early convalescence phase
Source: Res Sq. 2020 Aug 13:rs.3.rs-50023. Preprint. [Version 2] doi: 10.21203/rs.3.rs-50023/v2 (PMC7430587; doi:10.21203/rs.3.rs-50023/v2)
Supplement: Supplement [file SupplementaryTable2Revised.docx]

**Supplementary Table 2: Gross lung lesion severity scores in AGMs infected with SARS-CoV-2**

|  | **LUNGS (Gross Grades)** | | | | | | **Gross Score*** |
| --- | --- | --- | --- | --- | --- | --- | --- |
| **Subject No.** | **Right Upper Lobe (RUL)** | **Right Middle Lobe (RML)** | **Right Lower Lobe (RLL)** | **Left**  **Upper Lobe (LUL)** | **Left Middle Lobe (LML)** | **Left**  **Lower Lobe (LLL)** |  |
| **AGM-1** | 0 | 0 | 2 | 0 | 0 | 1 | 0.75 |
| **AGM-2** | 1 | 1 | 1 | 1 | 2 | 3 | 1.625 |
| **AGM-3** | 1 | 1 | 2 | 1 | 2 | 3 | 1.875 |
| **AGM-4** | 1 | 1 | 2 | 1 | 1 | 2 | 1.5 |
| **AGM-5** | 1 | 0 | 2 | 1 | 1 | 3 | 1.625 |
| **AGM-6** | 2 | 2 | 3 | 1 | 2 | 2 | 2.125 |
|  |  |  |  |  |  |  |  |

0 0%

1 1-25%

2 26-50%

3 51-75%

4 76-100%

* $\left[ \frac{\left( \frac{RUL+RML+LUL+LML}{4} \right)+\left( \frac{RLL+LLL}{2} \right)}{2} \right]=Gross Score$
